# Machine learning approaches to identify systemic lupus erythematosus in anti-nuclear antibody-positive patients using genomic data and electronic health records

**DOI:** 10.1186/s13040-023-00352-y

**Published:** 2024-01-05

**Authors:** Chih-Wei Chung, Seng-Cho Chou, Tzu-Hung Hsiao, Grace Joyce Zhang, Yu-Fang Chung, Yi-Ming Chen

**Affiliations:** 1 Department of Information Management, National Taiwan University, Taipei, Taiwan; 2 Department of Medical Research, Taichung Veterans General Hospital, Taichung, Taiwan; 3 Department of Public Health, Fu Jen Catholic University, New Taipei City, Taiwan; 4 Institute of Genomics and Bioinformatics, National Chung Hsing University, Taichung, Taiwan; 5 Department of Cellular and Physiological Sciences, The University of British Columbia, Vancouver, BC Canada; 6 Department of Electrical Engineering, Tunghai University, Taichung, Taiwan; 7 Division of Allergy, Immunology and Rheumatology, Department of Internal Medicine, Taichung Veterans General Hospital, 1650, Section 4, Taiwan Boulevard, Xitun Dist., Taichung City, 407 Taiwan; 8 Department of Post-Baccalaureate Medicine, College of Medicine, National Chung Hsing University, Taichung, Taiwan; 9 School of Medicine, College of Medicine, National Yang Ming Chiao Tung University, Taipei, Taiwan; 10 Rong Hsing Research Center for Translational Medicine & Ph.D. Program in Translational Medicine, National Chung Hsing University, Taichung, Taiwan; 11 Precision Medicine Research Center, College of Medicine, National Chung Hsing University, Taichung, Taiwan

**Keywords:** Machine learning, Systemic lupus erythematosus, Anti-nuclear antibody, Polygenic risk score, Single nucleotide polymorphism

## Abstract

**Background:**

Although the 2019 EULAR/ACR classification criteria for systemic lupus erythematosus (SLE) has required at least a positive anti-nuclear antibody (ANA) titer (≥ 1:80), it remains challenging for clinicians to identify patients with SLE. This study aimed to develop a machine learning (ML) approach to assist in the detection of SLE patients using genomic data and electronic health records.

**Methods:**

Participants with a positive ANA (≥ 1:80) were enrolled from the Taiwan Precision Medicine Initiative cohort. The Taiwan Biobank version 2 array was used to detect single nucleotide polymorphism (SNP) data. Six ML models, Logistic Regression, Random Forest (RF), Support Vector Machine, Light Gradient Boosting Machine, Gradient Tree Boosting, and Extreme Gradient Boosting (XGB), were used to identify SLE patients. The importance of the clinical and genetic features was determined by Shapley Additive Explanation (SHAP) values. A logistic regression model was applied to identify genetic variations associated with SLE in the subset of patients with an ANA equal to or exceeding 1:640.

**Results:**

A total of 946 SLE and 1,892 non-SLE controls were included in this analysis. Among the six ML models, RF and XGB demonstrated superior performance in the differentiation of SLE from non-SLE. The leading features in the SHAP diagram were anti-double strand DNA antibodies, ANA titers, AC4 ANA pattern, polygenic risk scores, complement levels, and SNPs. Additionally, in the subgroup with a high ANA titer (≥ 1:640), six SNPs positively associated with SLE and five SNPs negatively correlated with SLE were discovered.

**Conclusions:**

ML approaches offer the potential to assist in diagnosing SLE and uncovering novel SNPs in a group of patients with autoimmunity.

**Supplementary Information:**

The online version contains supplementary material available at 10.1186/s13040-023-00352-y.

## Introduction

Systemic Lupus Erythematosus (SLE) is a chronic autoimmune disease characterized by a wide range of clinical manifestations and diverse autoantibody profiles. Diagnosis of SLE is notoriously complex, often requiring a careful clinical evaluation and laborious serological testing [1]. The 2019 European League Against Rheumatism/American College of Rheumatology (EULAR/ACR) classification criteria for SLE stipulated the need for a positive anti-nuclear antibody (ANA) test with a titer equal to or exceeding 1:80 [2]. Despite this advancement, the precise identification of SLE patients continues to pose significant challenges for clinicians, mainly due to the heterogeneity of the disease and its overlap with other autoimmune diseases [3].

Over the past decade, there has been a substantial rise in the adoption of machine learning (ML) techniques in medical diagnosis, as they provide robust tools capable of deciphering intricate patterns and relationships within voluminous datasets [4, 5]. This computational approach has demonstrated significant utility in various clinical domains, from predicting disease outbreaks to personalizing treatment strategies [6, 7].

In the context of SLE, ML applications have shown promise in addressing several critical aspects of the disease, including refining diagnosis, identifying disease flares, predicting patient prognosis, and uncovering genetic and environmental risk factors. For instance, ML models have been utilized to identify potential biomarkers and create prediction models for disease activity, damage accrual, organ-specific involvement in SLE, and therapeutic response [8, 9]. Another study utilized ML algorithms to develop a risk probability index for SLE using clinical and demographic data [10]. Given the complexity and heterogeneity inherent in SLE diagnosis, applying ML techniques can significantly improve disease identification by utilizing both genomic data and electronic health records (EHRs). Consequently, these advancements can potentially revolutionize SLE management and research, contributing to improved patient outcomes [11].

Previously, we constructed an ML model to predict genomic susceptibility to SLE and rheumatoid arthritis (RA) [12]. That study also led to the discovery of genetic variations at the human leukocyte antigen (HLA) region crucial for differentiating RA from SLE. However, in clinical practice, rheumatologists generally diagnose SLE through the combination of an ANA test result and clinical feature assessment. Currently, no studies have reported the integration of ML models into the clinical diagnosis workflow of SLE. Therefore, this study aimed to fill this gap and explore the potential role of ML models in streamlining and improving the diagnosis of SLE.

This study aimed to employ six machine learning models—Logistic Regression (LR), Random Forest (RF), Support Vector Machine (SVM), Light Gradient Boosting Machine (LGBM), Gradient Tree Boosting (GTB), and Extreme Gradient Boosting (XGB)—to improve the identification of SLE patients using genomic data and EHRs from the Taiwan Precision Medicine Initiative (TPMI) cohort.

## Materials and methods

### Study population & study design

This study followed a retrospective case–control design utilizing data from the TPMI. The TPMI assembled EHRs and collected specimens from participants at the Taichung Veterans General Hospital (TCVGH), Taiwan, from June 2019 to June 2020, as previously described [12]. The analysis included participants who tested positive for ANA with a titer equal to or exceeding 1:80. Cases consisted of 946 patients diagnosed with SLE based on the 2012 Systemic Lupus International Collaborating Clinics classification criteria for SLE [13]. The control group, at a 1:2 ratio, was comprised of TPMI participants who tested positive for ANA but were not diagnosed with SLE. The study protocol received approval from the Ethics Committee of TCVGH (SF19153A), and all participants provided written informed consent.

### Genotyping

At TCVGH, we extracted DNA by automated platforms. Genotyping for each participant was conducted using the Taiwan Biobank version 2 (TWBv2) array, provided by Thermo Fisher Scientific, Inc. (Santa Clara, CA, USA). This array is specifically tailored for Genome-Wide Association Studies (GWAS), targeting known risk alleles. It encompasses a comprehensive set of 714,431 single nucleotide polymorphisms (SNPs), as delineated by Wei et al. in the prior research [14]. For optimal accuracy and to counteract potential batch inconsistencies, genotype calls were centrally processed by Academia Sinica in batches of 3,000 samples. In both cases and controls, the integrity of each SNP genotyping was assessed by evaluating the overall call rate (indicative of the successful call rate) and the minor allele frequency (MAF). Samples with call rates exceeding 95% were included in subsequent analyses. SNPs were excluded if they met any of the following conditions: If only one allele was present in both cases and controls, if the total call rate was below 95% or if the total MAF was less than 0.01, or if there was a significant departure from the Hardy–Weinberg equilibrium (*P* < 1 × 10^−4^).

### ANA test titers and patterns

ANA tests were conducted using an automated Indirect Immunofluorescence (IIF) NOVA View instrument and NOVA Lite HEp-2 ANA kit (Inova Diagnostics, Inc., San Diego, USA), as detailed by Wu et al*.* [15]. Titers and patterns of the ANA tests were reported in accordance with the expert-level reporting and interpretation principles stipulated by the International Consensus on ANA Patterns (ICAP) [16].

### Data extraction and preprocessing

The clinical parameters included a variety of demographic factors, comorbidities, baseline laboratory profiles, ANA titer and pattern profiles, and medication history. The index date was defined as the date of the initial ANA test with a titer equal to or exceeding 1:80, and the primary outcome was defined as the occurrence of SLE within one year following the index date. Comorbid conditions, such as diabetes mellitus, hypertension, and hyperlipidemia, were ascertained based on the patients' ICD codes registered prior to the index date. Baseline laboratory profiles, encompassing parameters such as anti-dsDNA antibody (Anti-dsDNA ab), C3, C4, white blood cell count (WBC), neutrophils, basophils, monocytes, eosinophils, hemoglobin, erythrocyte count, hematocrit, mean corpuscular volume, mean corpuscular hemoglobin, mean corpuscular hemoglobin concentration, erythrocyte distribution width, platelet count, serum creatinine, and estimated glomerular filtration rate (eGFR) [17], were evaluated within the year preceding and following the index date, and prior to the outcome date. ANA titers were observed at levels of 1:80, 1:160, 1:320, and ≥ 1:640, and ANA pattern profiles, including AC1, AC4, AC5, AC19, and AC24, were identified within six months after the index date and prior to the outcome date. Medication profiles encompassing glucocorticoids, hydroxychloroquine, cyclophosphamide, cyclosporin, mycophenolate mofetil, and azathioprine were ascertained using historical data extracted from EHRs during the six-month windows preceding and following the index date, and prior to the outcome date. Laboratory data features with missing value percentages exceeding 30% were excluded from the analysis. To address the missing values within the clinical features, the missForest imputation method was employed [18]. Continuous features were normalized using the RobustScaler technique, centering them around the median and scaling according to the interquartile range (IQR), thus ensuring that the ML models remained resilient against outliers [19]. For preprocessing the GWAS data, SNP values were encoded as 0, 1, or 2, representing the number of minor alleles under an additive genetic model [20]. Missing SNP values were imputed using the most frequent value within the training set. Additionally, the polygenic risk score (PRS) was computed using the candidate SNP features, serving as an assessment of individual genetic risk for ANA-positive patients who subsequently developed SLE [21]. The PRS for each SNP was calculated as follows:

$${{\text{PRS}}}_{tj}={\beta }_{j}\times {{\text{SNP}}}_{tj}$$

where *β*_*j*_ represents the effect size of the *j*^*th*^ SNP generated from the logistic regression, and *SNP*_*ij*_ is the feature value of the *j*^*th*^ SNP on the *i*^*th*^ patient.

However, the concept of the PRS did not incorporate the significance of the *p*-values derived from the association tests for pivotal SNPs. To address this limitation, a refined approach involving the aggregation of the adjusted PRS, which is weighted by the *p*-values obtained from the additive logistic regression, was introduced. This was executed separately for the top 50% of SNPs exhibiting significant positive and negative effects, yielding the following expressions:

$${{\text{PRSw}}}_{i}^{+}=\sum_{q=1}^{p}{\beta }_{q}\times {{\text{SNP}}}_{iq}\times {-{\text{log}}}_{10}\left({p-value}_{q}\right)$$

$${{\text{PRSw}}}_{i}^{-}=\sum_{q=1}^{p}{\beta }_{q}\times {{\text{SNP}}}_{iq}\times {-{\text{log}}}_{10}\left({p-value}_{q}\right)$$

where *p* and *n* are the total number of SNPs selected from the top 50% of significant *p*-values from the positive and negative effects, respectively.

### Feature selection

The initial step involved the application of the GWAS methodology to preselect SNPs exhibiting a strong association with ANA-positive patients diagnosed with SLE [20]. To discern the most pertinent SNP attributes, an association test was employed using the univariate logistic regression method [22]. Candidate SNPs were singled out based on a *p*-value threshold of less than 1 × 10^–3^, a measure taken to mitigate the effects of the high-dimensional nature inherent to GWAS analyses. To mitigate concerns related to overfitting, the default RF algorithm was employed to identify the top 5% most impactful SNPs for subsequent utilization as candidate features in the ensuing ML model construction process.

### Supervised Machine learning approaches

To forecast the likelihood of patients developing SLE within one year from the index date, six ML algorithms, LR, RF, SVM, LGBM, GTB, and XGB, were employed [11, 23]. The entire dataset (*n* = 2,838) was randomly divided into a training set (80%) and a testing set (20%), maintaining proportional representation across both sets. To optimize the ML algorithms' performance, hyperparameter optimization was employed. This optimization involved tuning the parameters using five-fold cross-validation and utilizing the GridSearchCV package within the training set. The validation set was used during the model training and optimization phases [24]. While fine-tuning the hyperparameters, the proposed models employ the training set to reach the optimized hyperparameters, without reference to the testing set. The optimized hyperparameters for each ML model are as described as Supplementary Table 1. Addressing the challenge of class imbalance, the Synthetic Minority Over-sampling Technique (SMOTE) was employed to balance the occurrences of the minority class [25]. Additionally, the TomekLinks method was implemented to regulate unnecessary instances of the majority class in the training set [25]. For feature interpretation and the pursuit of explainable artificial intelligence (XAI), the SHapley Additive exPlanations (SHAP) method was harnessed. This method enabled the identification of features closely associated with ANA-positive patients afflicted by SLE [26]. SHAP summary plots facilitated the visual representation of the relationship between feature values and the probability of the outcome. In order to robustly evaluate the performance of the ML algorithms in the context of binary classification with class imbalance, a set of metrics was employed. These metrics included accuracy, precision, sensitivity (recall), specificity, F1 score, Area Under the Receiver Operating Characteristic curve (AUROC), and Area Under the Precision-Recall curve (AUPRC). These metrics collectively gauged the efficacy of each classifier model [12]. To evaluate the robustness of the proposed algorithms, the statistical technique of bootstrapping-based resampling is employed to reconstruct the training dataset. Subsequently, these reconstructed sets are repeatedly trained by six ML models within a total of 500 iterations proposed in the phase. The average of 500-iteration training and validation is as quantified as the AUROC. The whole procedure consists of the predictive process and ML methodology, as illustrated in Supplementary Fig. 1.

### Statistical analysis

Continuous features are summarized as medians and their corresponding IQR, and their distributions were evaluated using a Wilcoxon rank-sum test. Binary features are represented as counts and percentages, and their associations were examined using either a Chi-square test or Fisher’s exact test, as appropriate. To uncover the relationships between ANA-positive patients and specific SNPs, as well as to elucidate the reasons for the absence of SLE development in patients with high-tier ANA (≥ 1:640), an association test was conducted. This test involved logistic regression analysis of SLE disease and SNPs in patients with an ANA titer equal to or exceeding 1:640. Univariate and multivariate logistic regression methods were employed to estimate the crude and adjusted odds ratios (aOR), accompanied by their corresponding 95% confidence intervals (CI). The data preprocessing and statistical analyses were executed using the R programming language (version 4.2.2), while the development of the ML models was carried out using Python (version 3.9.7). All statistical tests adhered to a two-sided configuration, with statistical significance set at *p*-values less than 0.05.

## Results

### Selection of candidate SNPs associated with SLE and non-SLE controls

**Fig. 1:**
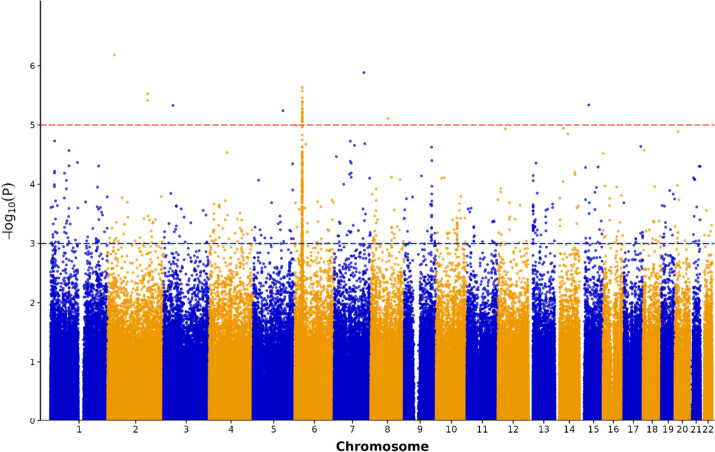
Manhattan plot for SLE obtained from GWAS results

Of the 686,438 imputed SNPs, the GWAS analysis identified a specific subset associated with SLE and non-SLE patients, as represented in the Manhattan plot (Fig. 1). Given the excessively stringent threshold of 1 × 10^–5^ (red line), the *p*-value threshold for selecting candidate genetic variants was adjusted to 1 × 10^–3^ (blue line), culminating in the selection of 684 SNPs. These SNPs were subsequently incorporated into the PRS calculation and ML model construction.

### Baseline characteristics of the study population

A total of 2,838 adults with available clinical data, SNP information, and ANA titers equal to or exceeding 1:80 was recruited for this study. Among them, 946 patients were diagnosed with SLE within one year following the index date (Table 1). The median age for the non-SLE and SLE patients was 45.3 and 42.6 years, respectively. Comparing the SLE and non-SLE patients, notable distinctions were observed. The SLE group exhibited a significantly lower prevalence of diabetes mellitus (*p* < 0.001), hypertension (*p* < 0.001), and hyperlipidemia (*p* < 0.001). Analysis of laboratory profiles revealed that the SLE patients had elevated levels of anti-dsDNA antibody, monocytes, mean corpuscular hemoglobin concentration, erythrocyte distribution width, and eGFR. Conversely, levels of C3, C4, WBC, basophils, eosinophils, hemoglobin, erythrocyte count, hematocrit, and platelets were notably higher in the non-SLE group. Regarding medication profiles, SLE patients had significantly higher usage rates of glucocorticoids, hydroxychloroquine, and azathioprine. However, the proportion of patients using cyclosporin was significantly lower in the SLE group than in the non-SLE group. Assessing the ANA titer results at the index date. The non-SLE group had a significantly greater proportion of ANA titers at 1:80 (*p* < 0.001) and at 1:160 and 1:320 (*p* = 0.002) compared with the SLE group. Nonetheless, the contrast between the non-SLE and SLE groups was evident in patients with ANA titers equal to or exceeding 1:640, with respective counts of 306 (16.2%) and 463 (48.9%). Analyzing the ANA pattern profiles, the non-SLE patients demonstrated higher proportions for AC1, AC4, and AC24, whereas AC5 exhibited an inverse trend. Additionally, when examining the median values of the PRS, PRSw + , and PRSw-, all three were significantly higher in the SLE group compared with the non-SLE group: PRS (8.6, IQR: -7.6 to 22.7), PRSw + (85.3, IQR: 59.9 to 118.5), and PRSw- (-57.2, IQR: -90.2 to -42.5).

**Table 1:** Baseline demographic and clinical characteristics of the study population

Variables	ALL (*n* = 2838)	Non-SLE (*n* = 1892)	SLE (*n* = 946)	*p*-value^a^
Age at index date (year)	44.5 (33.7, 56.4)	45.3 (34.6, 57.3)	42.6 (31.9, 55.2)	< 0.001
Male, *n* (%)	348 (12.3)	232 (12.3)	116 (12.3)	1
Comorbidity, *n* (%)				
Diabetes mellitus	235 (8.3)	186 (9.8)	49 (5.2)	< 0.001
Hypertension	415 (14.6)	321 (17.0)	94 (9.9)	< 0.001
Hyperlipidemia	331 (11.7)	261 (13.8)	70 (7.4)	< 0.001
Laboratory profiles, median (IQR)				
Anti-dsDNA ab (WHO unit/ml)	47.7 (22.1, 131.1)	32.9 (16.3, 66.3)	123.5 (42.1, 450.3)	< 0.001
C3 (mg/dl)	110.2 (93.3, 128.0)	115.9 (100.4, 132.0)	97.6 (78.0, 114.9)	< 0.001
C4 (mg/dl)	25.1 (18.5, 32.3)	27.4 (21.3, 34.3)	19.2 (12.7, 26.3)	< 0.001
WBC (/mm^3^)	6200 (5010, 7900)	6430 (5260, 8063)	5680 (4500, 7445)	< 0.001
Neutrophils (%)	63.8 (56.5, 72.0)	63.4 (56.5, 71.5)	64.6 (56.3, 73.3)	0.131
Basophils (%)	0.5 (0.3, 0.7)	0.5 (0.3, 0.7)	0.4 (0.3, 0.6)	< 0.001
Monocytes (%)	6.0 (4.8, 7.7)	5.9 (4.8, 7.2)	6.5 (5.0, 8.7)	< 0.001
Eosinophils (%)	1.5 (0.7, 2.7)	1.7 (0.9, 2.9)	1.2 (0.5, 2.4)	< 0.001
Hemoglobin (g/dl)	12.7 (11.6, 13.7)	12.9 (11.8, 13.8)	12.4 (11.3, 13.5)	< 0.001
Erythrocyte (10^6^/μL)	4.3 (3.9, 4.7)	4.3 (4.0, 4.7)	4.2 (3.8, 4.6)	< 0.001
Hematocrit	38.4 (35.0, 41.0)	38.7 (35.5, 41.2)	37.3 (34.0, 40.2)	< 0.001
Mean corpuscular volume	89.8 (85.8, 93.1)	89.9 (85.9, 93.1)	89.5 (85.3, 93.0)	0.531
Mean corpuscular hemoglobin	30.0 (28.4, 31.3)	30.0 (28.4, 31.2)	30.0 (28.4, 31.5)	0.189
Mean corpuscular hemoglobin concentration	33.3 (32.4, 34.0)	33.2 (32.3, 33.9)	33.3 (32.5, 34.1)	0.001
Erythrocyte distribution width	13.2 (12.5, 14.4)	13.2 (12.5, 14.3)	13.5 (12.7, 14.7)	< 0.001
Platelets (/mm^3^)	250 (202, 301)	259 (210, 310)	233 (185, 284)	< 0.001
Creatinine (mg/dL)	0.7 (0.6, 0.9)	0.7 (0.7, 0.9)	0.7 (0.6, 0.9)	0.129
eGFR (mL/min/1.73 m^2^)	77.5 (65.3, 90.8)	77.2 (65.1, 90.2)	78.4 (65.4, 92.8)	0.018
Medication profiles, *n* (%)				
Glucocorticoid	1616 (56.9)	970 (51.3)	646 (68.3)	< 0.001
Hydroxychloroquine	1714 (60.4)	960 (50.7)	754 (79.7)	< 0.001
Cyclophosphamide	98 (3.5)	71 (3.8)	27 (2.9)	0.217
Cyclosporin	153 (5.4)	114 (6.0)	39 (4.1)	0.034
Mycophenolate mofetil	74 (2.6)	56 (3.0)	18 (1.9)	0.096
Azathioprine	322 (11.3)	163 (8.6)	159 (16.8)	< 0.001
ANA titer at index date, *n* (%)				
1:80	845 (29.8)	731 (38.6)	114 (12.1)	< 0.001
1:160 & 1:320	1224 (43.1)	855 (45.2)	369 (39.0)	0.002
≥ 1:640	769 (27.1)	306 (16.2)	463 (48.9)	< 0.001
ANA pattern profiles, *n* (%)				
AC1	1114 (39.3)	799 (42.2)	315 (33.3)	< 0.001
AC4	1824 (64.3)	1374 (72.6)	450 (47.6)	< 0.001
AC5	124 (4.4)	60 (3.2)	64 (6.8)	< 0.001
AC19	110 (3.9)	72 (3.8)	38 (4.0)	0.783
AC24	91 (3.2)	86 (4.5)	5 (0.5)	< 0.001
Polygenic risk profiles, median (IQR)				
PRS	-2.6 (-18.1, 12.3)	-7.7 (-21.7, 6.4)	8.6 (-7.6, 22.7)	< 0.001
PRSw +	70.2 (48.2, 102.9)	64.3 (44.8, 93.0)	85.3 (59.9, 118.5)	< 0.001
PRSw-	-71.3 (-100.5, -51.5)	-77.7 (-105.1, -56.7)	-57.2 (-90.2, -42.5)	< 0.001

Anti-dsDNA ab: anti-dsDNA antibody; WBC: white blood cell; eGFR: estimated glomerular filtration rate; PRS: polygenic risk score; PRSw+ and PRSw-: modified PRS weighted by the *p*-value from SNPs among the top-50% positive and negative effect, respectively

^a^*p*-values were calculated by Wilcoxon rank-sum test for continuous variables and Chi-square test (or Fisher’s exact test as appropriate) for categorical variables

### Comparison of model performance on the unseen testing set

**Fig. 2:**
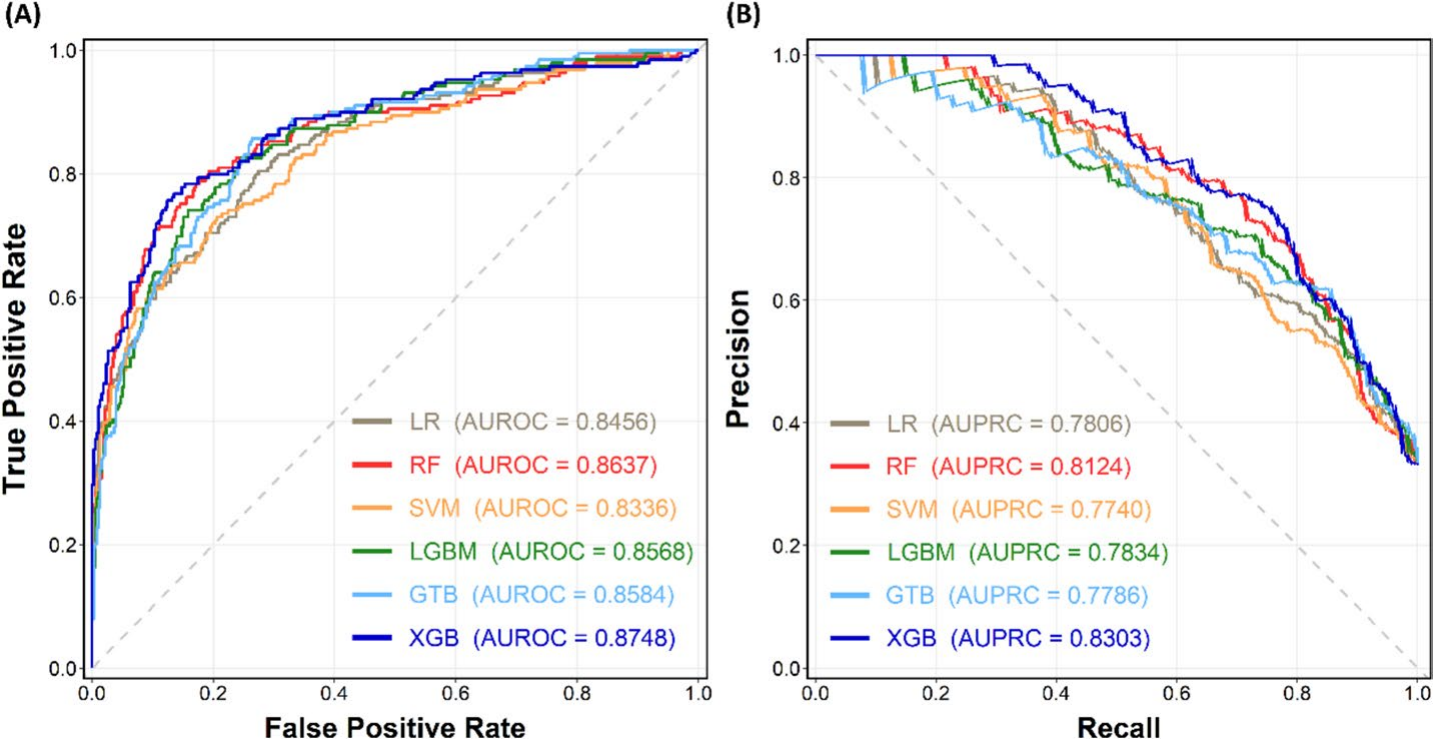
Performance evaluation of the six proposed ML models. (**A**) ROC curve and (**B**) PR curve

Table 2 presents the evaluation of the performance of six distinct ML algorithms on the unseen testing set using various metrics. Given the class imbalance within this study, particular attention was directed toward the assessment metrics of F1 score, AUROC, and AUPRC. The computed AUROC values consistently exceeded 0.8 across all six models, indicating favorable discrimination ability. Notably, the XGB and RF methodologies exhibited superior performance in the F1 score and AUPRC metrics. For a comprehensive depiction, the ROC and PR curves are presented in Fig. 2. Impressively, the XGB model had the highest performance, with an AUROC of 0.8748 and an AUPRC of 0.8303. The RF model was also noteworthy and yielded commendable results with an AUROC of 0.8637 and an AUPRC of 0.8124. Both the five-fold cross-validation and bootstrapping validation methods reach similar results, with a 95% CI for the AUROC, as shown in Supplementary Table 2.

**Table 2:** Model Performance of the proposed ML models on the unseen testing set

Classifier	Accuracy	Precision	Sensitivity	Specificity	F1 score	AUROC	AUPRC
LR	0.7887	0.6949	0.6508	0.8575	0.6721	0.8456	0.7806
RF	0.8345	0.7746	0.7090	0.8971	0.7403	0.8637	0.8124
SVM	0.7729	0.6429	0.7143	0.8021	0.6767	0.8336	0.7740
LGBM	0.7993	0.7193	0.6508	0.8734	0.6833	0.8568	0.7834
GTB	0.7975	0.7033	0.6772	0.8575	0.6900	0.8584	0.7786
XGB	0.8345	0.7684	0.7196	0.8918	0.7432	0.8748	0.8303

### Interpretation of the risk factors associated with SLE in the ML models

**Fig. 3:**
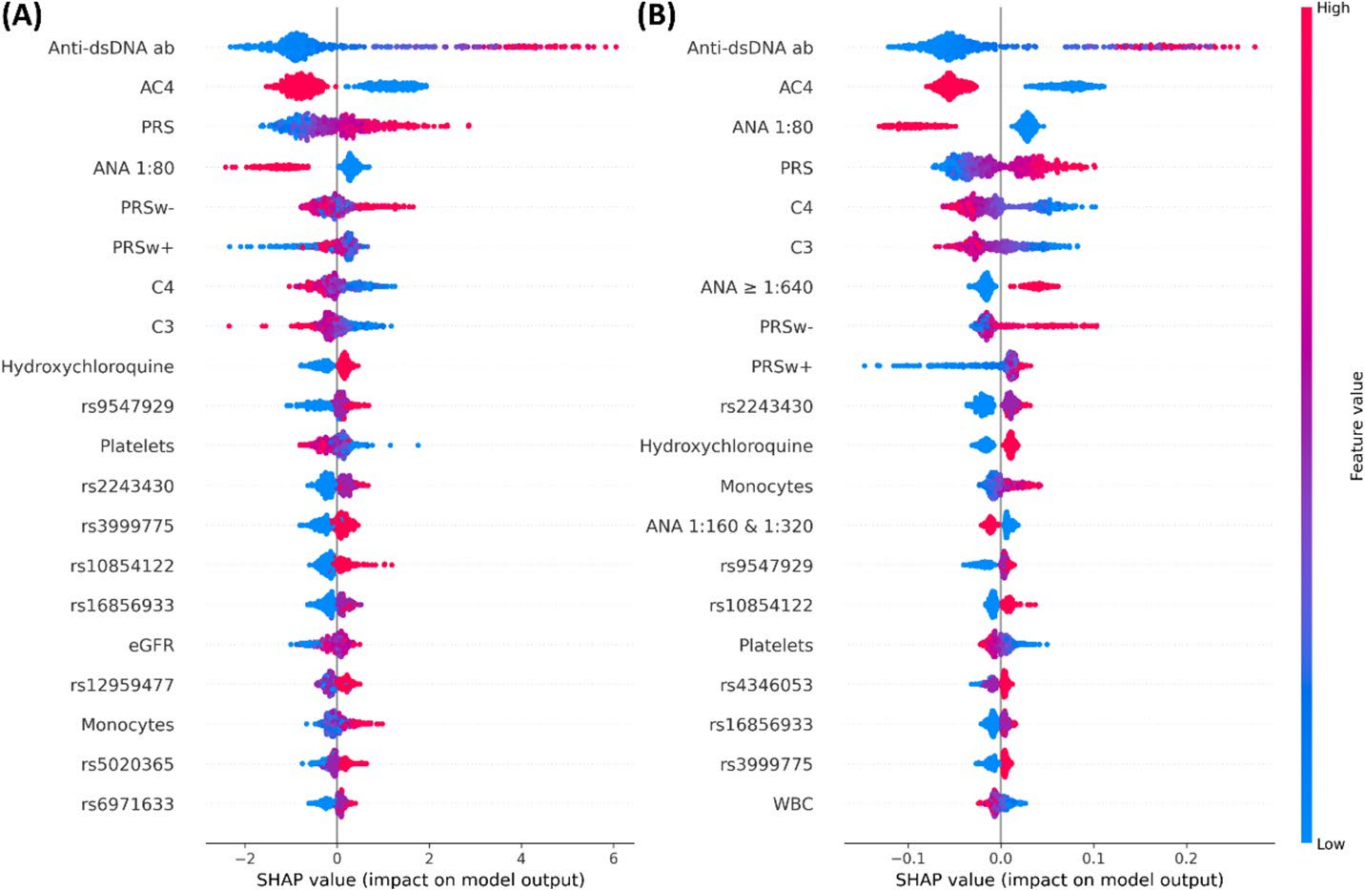
SHAP values of the top 20 features for identifying ANA-positive patients with SLE. (**A**) XGB model and (**B**) RF model

To illuminate the contributions of features to the proposed ML models, the SHAP summary plot was employed, displaying the top 20 risk factors (Fig. 3). An upward trend in the SHAP value of a feature corresponds to an increased likelihood of developing SLE within one year. Notably, the features anti-dsDNA ab, AC4, PRS, and ANA titer 1:80 exhibited similar trends and had the highest importance within the XGB and RF models. Furthermore, the SHAP summary values provide novel insights into the significance of individual SNP features. For instance, ANA-positive patients had a comparatively elevated risk of developing SLE within one year when their SNP features, such as rs9547929, rs2243430, and rs16856933, carried genotypes of 0/1 or 1/1. This information offers valuable understanding regarding the genetic factors associated with the development of SLE in ANA-positive patients.

### Association between SLE and SNPs among patients with high titer ANA

Clinically high-titer ANA (≥ 1:640) is associated with a diagnosis of SLE. Further logistic regression analyses were conducted on SNPs that showed positive and negative correlations with SLE in the subgroup of participants with high-titer ANA (Table 3). Six SNPs (rs13029062, rs71909377, rs62175694, rs13268741, rs6971633, and rs16905611) positively correlated with an SLE outcome were identified. In addition, five SNPs (rs6455889, rs9910586, rs712735, rs5020365, and rs4346053) were discovered as protective factors against SLE in the high-titer ANA group.

**Table 3:** Association between SLE and SNPs among patients with high-titer ANA (≥ 1:640) at index date

Gene	SNP	SNP value	*n*	Crude OR (95% CI)	Adjusted OR (95% CI)^a^
VIT	rs13029062	0	429	Reference	Reference
		1	285	1.24 (0.91, 1.68)	1.32 (0.95, 1.82)
		2	55	3.06 (1.59, 6.38)**	3.00 (1.52, 6.39)**
	rs71909377	0	343	Reference	Reference
		1	342	1.38 (1.02, 1.87)*	1.49 (1.08, 2.06)*
		2	84	2.19 (1.31, 3.76)**	2.53 (1.49, 4.44)***
	rs62175694	0	550	Reference	Reference
		1	201	1.54 (1.10, 2.17)*	1.54 (1.08, 2.21)*
		2	18	2.63 (0.93, 9.37)	2.47 (0.82, 9.42)
SLC7A2	rs13268741	0	232	Reference	Reference
		1	388	1.41 (1.01, 1.95)*	1.43 (1.01, 2.02)*
		2	149	1.92 (1.25, 2.97)**	1.94 (1.24, 3.06)**
AUTS2	rs6971633	0	259	Reference	Reference
		1	382	1.53 (1.11, 2.11)**	1.64 (1.17, 2.31)**
		2	128	1.44 (0.94, 2.23)	1.49 (0.95, 2.36)
	rs16905611	0	335	Reference	Reference
		1	344	1.57 (1.15, 2.14)**	1.58 (1.15, 2.19)**
		2	90	1.45 (0.90, 2.36)	1.35 (0.82, 2.25)
PACRG	rs6455889	0	324	Reference	Reference
		1	357	0.92 (0.68, 1.26)	0.97 (0.70, 1.34)
		2	88	0.51 (0.32, 0.82)**	0.53 (0.32, 0.88)*
	rs9910586	0	438	Reference	Reference
		1	289	0.81 (0.60, 1.10)	0.81 (0.59, 1.11)
		2	42	0.48 (0.25, 0.92)*	0.49 (0.25, 0.95)*
	rs712735	0	324	Reference	Reference
		1	353	0.85 (0.62, 1.16)	0.78 (0.56, 1.08)
		2	92	0.63 (0.40, 1.01)	0.59 (0.36, 0.97)*
	rs5020365	0	245	Reference	Reference
		1	365	0.74 (0.53, 1.04)	0.76 (0.53, 1.08)
		2	159	0.50 (0.33, 0.75)***	0.50 (0.32, 0.77)**
	rs4346053	0	414	Reference	Reference
		1	296	0.69 (0.51, 0.94)*	0.72 (0.52, 0.99)*
		2	59	0.71 (0.41, 1.23)	0.72 (0.40, 1.29)

^a^Adjusted for age, sex, diabetes, hypertension, and hyperlipidemia; * *p* < 0.05, ** *p* < 0.01, *** *p* < 0.001

### Prediction of SLE in selected patient populations by ML algorithm

**Fig. 4:**
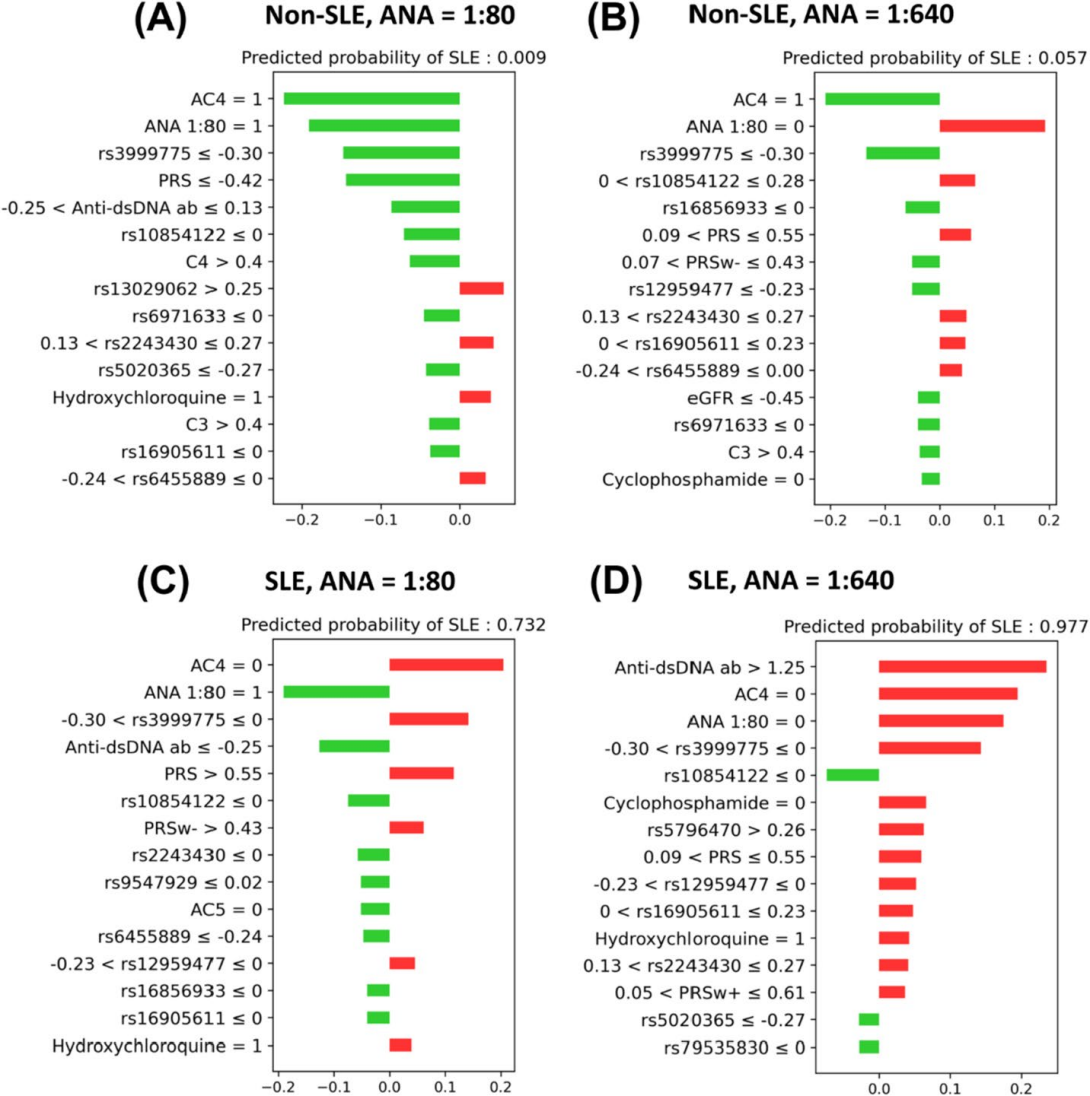
Predicted probability of SLE in selected patient populations with low titer (1:80, A and C) and high titer (1:640, B and D) ANA by XGB model

Figure 4 delineates the efficacy of the ML model in stratifying selected patients into  those with and without SLE based on ANA titers, ANA patterns, SNPs, PRS, and relevant clinical features. As evident in Fig. 4 (A) and (D), patients with ANA titers of 1:80 and 1:640 manifest predicted probabilities of SLE at 0.009 and 0.977, respectively, when factoring in the cumulative influence of ANA patterns, SNPs, PRS, and clinical features that are either suggestive or contraindicative of SLE diagnosis. Conversely, Fig. 4 (B) showcases the capacity of the ML model to accurately negate the diagnosis of SLE in patients presenting with high-titer ANA (1:640), taking into account corresponding ANA patterns, SNPs, PRSw, and clinical features. Similarly, Fig. 4 (C) depicts a scenario where the ML model correctly identified a patient with a low-titer ANA (1:80) as having SLE, based on ANA patterns, SNPs, PRS, PRSw, and pertinent clinical features.

## Discussion

This study is the first to construct ML models for the identification of SLE patients from a cohort with positive ANA tests, incorporating genome-wide SNPs, PRS, and clinical features. Autoantibody profiles, ANA patterns, complement levels, and genetic variations were ascertained as principal contributing factors for SLE. Concurrently, in the subgroup with high-titer ANA, a characteristic indicative of SLE diagnosis, SNPs positively and negatively associated with SLE were discerned. These results shed light on the potential for integrating ML models into the diagnostic workflow for systemic autoimmune disease, fostering a more precise, comprehensive, and data-informed approach to patient diagnosis and care.

ML models have been extensively explored for diagnosing SLE, defining clinical phenotypes, determining outcomes, and informing therapeutic decisions [23]. Previous studies utilizing ML to facilitate SLE diagnosis have employed diverse input data, including EHRs, genetic biomarkers, proteomics, lipidomes, or a combination of these data types [23, 24, 27–32]. This study is the first to incorporate genome-wide SNPs, PRS, and EHRs in an ML analysis. Moreover, ML algorithms for diagnostic purposes in previous studies included RF, LASSO, SVM, LR, XGB, and Partial Least Square [23, 24, 27–32]. This study is the first to attempt to compare the diagnostic accuracy among six ML models. In line with the classification criteria proposed by EULAR/ACR, this study is novel in integrating the ANA test as the primary criterion for cohort enrollment. This study also included the ANA titer and ANA pattern in the input data for the ML models. These innovative aspects of this research pave the way for potential clinical applications, particularly for rheumatologists encountering patients presenting with autoimmune features and a positive ANA test. We postulate that ANA patterns, PRS, SNPs, and autoantibody profiles could provide additional diagnostic insights for SLE. Future investigations are necessary to validate these findings and further explore the potential of this integrated ML approach in diagnosing and managing SLE.

This study identified six SNPs positively associated with SLE and five SNPs negatively associated with SLE in participants with high-titer ANAs. The *VIT* gene, which is involved in iron transportation, metabolism, and antioxidant protein catalase activity, was among these [33]. Another gene, SLC7A2, encodes a cationic amino acid transporter and has been reported to be associated with inflammatory responses in asthma [34]. The *AUTS2* gene, implicated in the neurodevelopmental process and acute lymphoblastic leukemia, was also noted [35, 36]. Lastly, the *PACRG* gene, linked with Parkinson’s disease and increased susceptibility to leprosy, was identified [37, 38]. Notably, none of these genes have been previously reported in relation to SLE. This study, therefore, presents a potential approach to discovering novel genetic variants associated with autoimmune diseases. Future research is needed to clarify the mechanistic associations between these genes and autoimmunity, further enhancing the understanding of the genetic underpinnings of such diseases.

To delve into potential risk factors and achieve optimal performance, particularly within the context of an imbalanced dataset, the employment of an advanced ensemble ML framework was a judicious choice. Additionally, XAI was applied to assess SLE disease risk and select pivotal SNP features for predictive modeling. Prior research has demonstrated that incorporating SNP and PRS features can substantially enhance disease prediction accuracy [12]. The notion of PRS, encompassing the cumulative effects of numerous candidate SNP features, offers invaluable insights for detecting complex diseases and identifying high-risk patients [21]. However, traditional computation methods of PRS [21, 39, 40] neglect variations of significance of *p*-value and directional attributes of SNP features [41, 42]. By integrating potential SNPs, the PRS makes the importance of cumulative effect sizes specific. Based on the magnitude of PRS, such as high or low quartiles, we can establish an association with disease progression [42, 43]. Consequently, two novel features resulted from the adjusted PRS are formulated; by the features, the accuracy of predicts for case group and control group can be greatly enhanced. Figure 3 illustrates the outcomes, revealing that control and case patients can be discerned by the PRSw + and PRSw- features, respectively. Notably, this distinction holds even in the context of enrolling ANA-positive patients, which might lead to a somewhat homogenous population within the study.

Despite being the first study to integrate the ANA test and SLE classification workflow as enrollment criteria, this research has several limitations. First, the study design is retrospective, which inevitably leads to incomplete data in the EHRs. Additionally, structured assessments for autoimmune clinical features were not prospectively collected, which might have affected the richness of the input data. Secondly, the input data for the ML models did not encompass cytokine, transcriptomic, or proteomic datasets, thereby possibly limiting the breadth of these mechanistic interpretations. Lastly, the study cohort was exclusively composed of Taiwanese-ethnic Chinese participants. As such, the results may not be universally applicable, restricting their extrapolation to populations of other ancestries.

In conclusion, this study establishes a novel application of ML models for the diagnosis of SLE using genomic and clinical data. The integration of ANA tests, genomic data of SNPs and PRS, and clinical features offer a promising approach for identifying patients with SLE, thereby potentially improving diagnostic precision. Moreover, this research introduces a possible method for discovering novel genetic variants associated with autoimmune diseases. This study offers an encouraging step toward integrating ML into the diagnostic workflow for systemic autoimmune diseases.

### Supplementary Information

**Additional file 1. **

**Additional file 2. **

## Data Availability

Data is available upon reasonable request to the corresponding author with a statistical analysis plan.
