# Supplementary material for: Machine learning approaches to identify systemic lupus erythematosus in anti-nuclear antibody-positive patients using genomic data and electronic health records
Source: BioData Min. 2024 Jan 5;17:1. doi: 10.1186/s13040-023-00352-y (PMC10770905; doi:10.1186/s13040-023-00352-y)
Supplement: Supplementary file 1 — Additional file 1. [file 13040_2023_352_MOESM1_ESM.docx]

Supplementary Table 1 Optimal hyperparameter settings of six proposed models

| Model | Hyperparameter | Search space | Optimal hyperparameter |
| --- | --- | --- | --- |
| LR | C | {0.001, 0.0025, 0.005, …, 13, 14, 15} | 0.01 |
|  | penalty | {l1, l2} | l1 |
|  | class_weight | {auto, balanced, None} | balanced |
|  | solver | {newton-cg, lbfgs, liblinear, sag, saga} | saga |
| RF | n_estimators | {100, 150, 200, …, 450, 500} | 150 |
|  | criterion | {gini, entropy} | gini |
|  | max_depth | {1, 3, 5, …, 25} | 23 |
|  | min_samples_split | {1, 2, 3, …, 9} | 5 |
|  | min_samples_leaf | {1, 2, 3, …, 9} | 1 |
|  | class_weight | {balanced, balanced_subsample, None} | balanced |
| SVM | kernel | {linear, poly, rbf, sigmoid} | rbf |
|  | C | {1, 2, 3, …, 150} | 6 |
|  | gamma | {1e-05, 5e-05, 1e-04, …, 1e-02} | 5e-05 |
|  | degree | {1, 2, …, 10} | 2 |
|  | tol | {1e-05, 5e-05, 1e-04, …, 1e-02} | 1e-05 |
| LGBM | boosting_type | {gbdt, dart, goss} | gbdt |
|  | learning_rate | {0.001, 0.005, …, 0.2, 0.25} | 0.15 |
|  | n_estimators | {100, 150, …, 500} | 150 |
|  | num_leaves | {1, 3, …, 13, 15} | 9 |
|  | max_depth | {1, 3, …, 13, 15} | 7 |
|  | class_weight | {balanced, None} | balanced |
|  | min_child_samples | {1, 3, …, 13, 15} | 3 |
|  | min_child_weight | {1e-04, 5e-04, …, 1e-02} | 5e-03 |
|  | subsample | {0.7, 0.8, 0.9, 1.0} | 0.7 |
| GTB | n_estimators | {50, 100, …, 450, 500} | 300 |
|  | learning_rate | {0.001, 0.05, ..., 0.35, 0.4} | 0.3 |
|  | max_depth | {1, 3, …, 15} | 3 |
|  | min_samples_leaf | {1, 3, …, 15} | 11 |
|  | min_samples_split | {1, 3, …, 15} | 3 |
|  | max_leaf_nodes | {1, 3, …, 15} | 5 |
|  | max_features | {40, 50 ,60 ,70} | 60 |
|  | tol | {1e-06, 5e-06, …, 1e-02} | 1e-05 |
| XGB | booster | {gbtree, gblinear, dart} | gbtree |
|  | learning_rate | {0.001, 0.05, ..., 0.45, 0.5} | 0.4 |
|  | n_estimators | {50, 100, …, 450, 500} | 50 |
|  | max_depth | {1, 3, …, 19} | 15 |
|  | max_leaves | {1, 3, …, 9} | 1 |
|  | gamma | {0, 0.0001, …, 0.1} | 0.2 |
|  | min_child_weight | {1e-06, 5e-06, …, 1e-02} | 1e-06 |
|  | subsample | {0.7, 0.8, 0.9, 1.0} | 0.8 |

Supplementary Table 2 Comparison of ML models in AUROC and 95% CI through two validated approaches

| Model | Five-fold cross-validation | Bootstrap sampling |
| --- | --- | --- |
| LR | 0.8633 (0.8373, 0.8892) | 0.8139 (0.8028, 0.8243) |
| RF | 0.9463 (0.9281, 0.9645) | 0.9418 (0.9282, 0.9547) |
| SVM | 0.8609 (0.8300, 0.8918) | 0.8302 (0.8230, 0.8363) |
| LGBM | 0.9423 (0.9271, 0.9574) | 0.9325 (0.9152, 0.9492) |
| GTB | 0.9422 (0.9280, 0.9565) | 0.9322 (0.9155, 0.9488) |
| XGB | 0.9403 (0.9228, 0.9578) | 0.9357 (0.9186, 0.9504) |


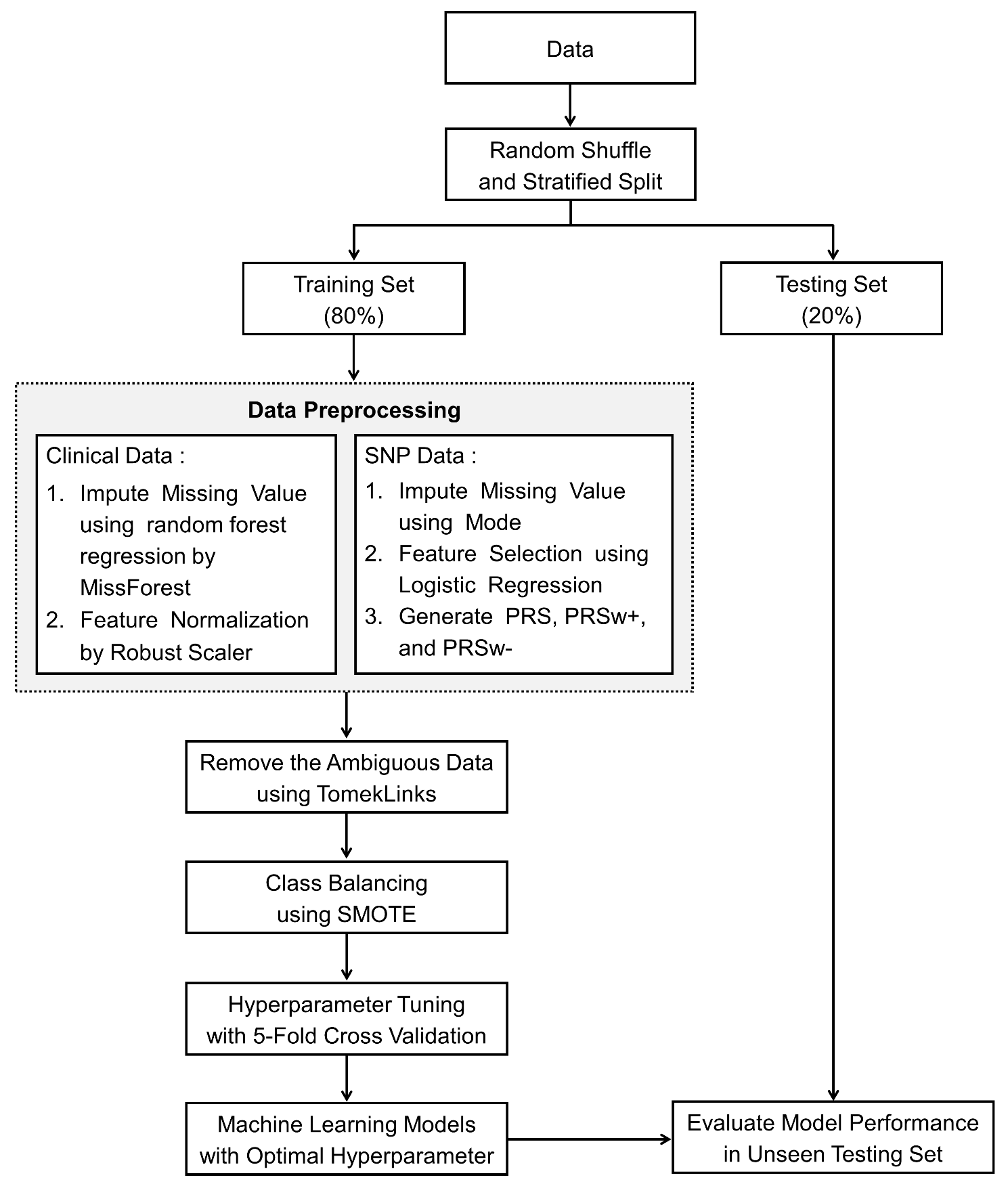
 Supplementary Fig. 1 The diagram of the predictive process for SLE in ANA-positive patients
