# Supplementary figures and images for: Machine learning approaches to identify systemic lupus erythematosus in anti-nuclear antibody-positive patients using genomic data and electronic health records
Source: BioData Min. 2024 Jan 5;17:1. doi: 10.1186/s13040-023-00352-y (PMC10770905; doi:10.1186/s13040-023-00352-y)

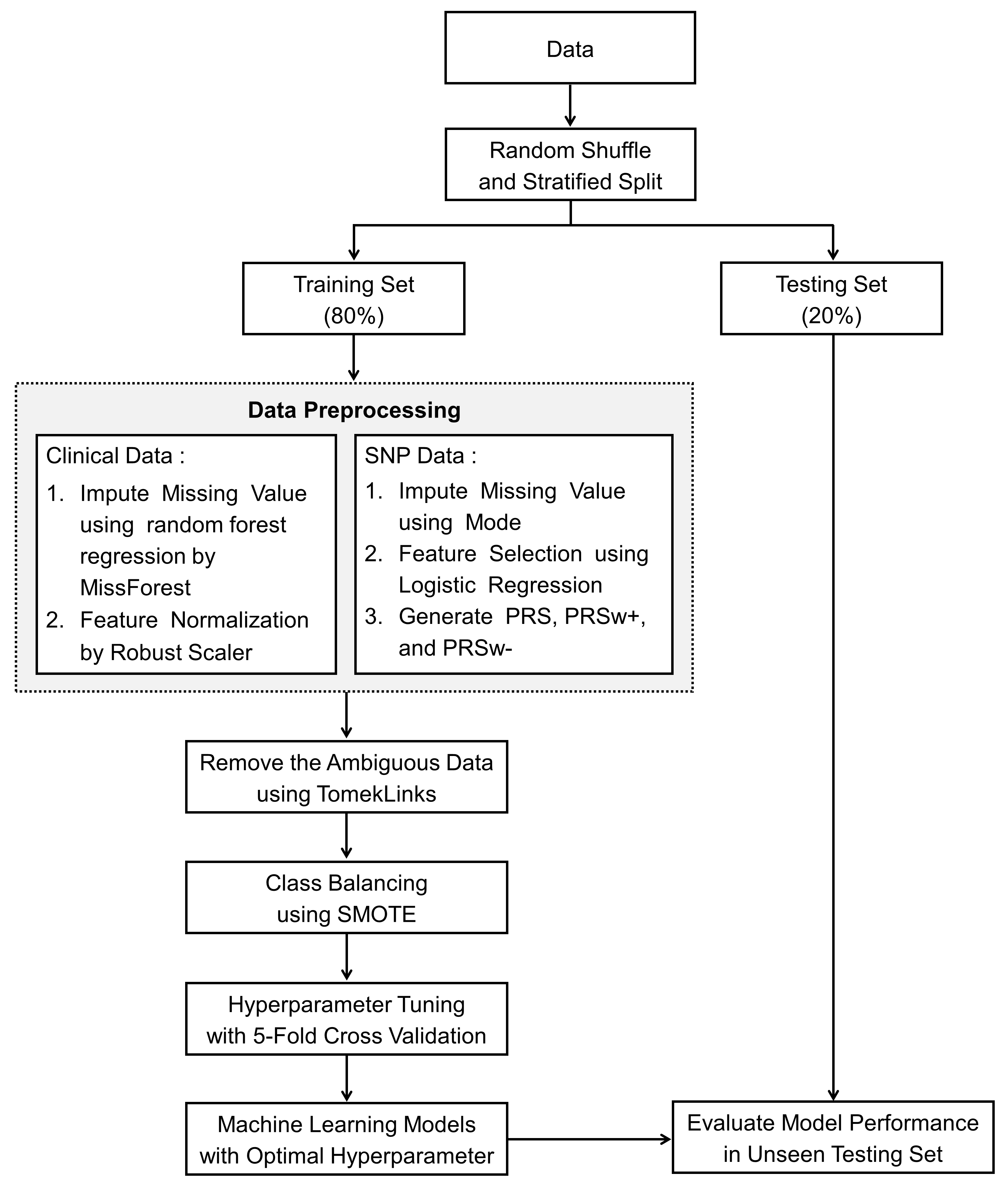

Supplement: Supplementary file 2 — Additional file 2. [file 13040_2023_352_MOESM2_ESM.tif]
